# Conceptual and Visual Features Contribute to Visual Memory for Natural Images

**DOI:** 10.1371/journal.pone.0037575

**Published:** 2012-06-13

**Authors:** Gesche M. Huebner, Karl R. Gegenfurtner

**Affiliations:** Department of Psychology, Justus-Liebig-University of Giessen, Giessen, Germany; University of Regensburg, Germany

## Abstract

We examined the role of conceptual and visual similarity in a memory task for natural images. The important novelty of our approach was that visual similarity was determined using an algorithm [1] instead of being judged subjectively. This similarity index takes colours and spatial frequencies into account. For each target, four distractors were selected that were (1) conceptually and visually similar, (2) only conceptually similar, (3) only visually similar, or (4) neither conceptually nor visually similar to the target image. Participants viewed 219 images with the instruction to memorize them. Memory for a subset of these images was tested subsequently. In Experiment 1, participants performed a two-alternative forced choice recognition task and in Experiment 2, a yes/no-recognition task. In Experiment 3, testing occurred after a delay of one week. We analyzed the distribution of errors depending on distractor type. Performance was lowest when the distractor image was conceptually and visually similar to the target image, indicating that both factors matter in such a memory task. After delayed testing, these differences disappeared. Overall performance was high, indicating a large-capacity, detailed visual long-term memory.

## Introduction

It is a well-established finding that long-term memory for images is extensive and that thousands of items can be stored [2]–[5]. For example, after seeing 2560 slides for 10 seconds each participants’ recognition performance, even after several days, was still above 90% [5]. Even when 10,000 images were shown [4], participants were able to decide with an accuracy of 83% which of two images they had seen. However, this remarkable ability to successfully discriminate between targets and distractors did not necessarily mean that a detailed representation of each image was created and stored. Distractor and target images were rather dissimilar in those studies, possibly allowing participants to rely upon merely storing the basic meaning of an image, in terms of a short verbal description. Thus, to evaluate what such a seemingly remarkable memory performance means, a measure of the level of stored detail would be needed.

Research from the field of change detection suggests that our stored representations lack visual detail. Large changes in scenes can go unnoticed when the change occurs during some sort of perceptual disruption, for example an ISI [6], an eye movement [7]–[10] or an occlusion [11]. The last study mentioned is one of the most dramatic examples for change blindness: an experimenter asked a pedestrian for directions, and during the conversation, an interruption was created by having a door carried in between pedestrian and experimenter. While the experimenter was occluded, he was replaced by a different person. Only half of the pedestrians detected the change. Thus, the concept of change blindness provides arguments that the visual system does not build a complete representation of a scene encompassing all details. Recognition memory for highly similar stimuli such as crystal pattern of snowflakes was also comparatively low [12]. In turn, this suggests that in the earlier experiments [3]–[5], no detailed representations about the thousand images were created but instead rather abstract, categorical information was stored.

However, evidence exists that more than only the gist of an image is stored in visual memory [13]–[17]. When visual memory was tested by pairing the target object either with a distractor from the same basic-category (a token change) or with a rotated version of the original target [14], a low discrimination performance would have indicated that participants had only remembered the conceptual meaning; however, it was very high (mean percentage correct were 85% and 92%, respectively). Reliably above-chance discrimination performance for token changes was even observed when more than 400 objects had been seen between the initial viewing of an object and the test [13]. A recent study demonstrated that long-term memory is capable of storing a massive number of objects in great detail [15]. After viewing of 2,500 objects, participants were tested on three types of target-distractor pairings. The previously seen object was either paired with an object from a novel category, an object of the same basic level category, or with the same object only in a different state. For the first condition, remembering the gist would have been enough for correct discrimination whereas in the second condition, storing only the basic category would have led to chance performance. In the supposedly even harder third condition, details about the identity and state of the object needed to be remembered. Performance was very high in all of these conditions with mean percentages correct of 92%, 88%, and 87%, respectively. This suggests that detailed representations of thousands of objects can be stored and that participants extract many visual details in addition to a conceptual description of an image. Similar results were found for the memorization of scenes [16]. Observers saw a large number of scenes and were then presented with a 2-AFC recognition task where the foil either came from the same basic category as the target or from a different category. Whilst performance was significantly higher for pairings with a novel object, observers were able to correctly recognize the target image in 84% of trials even when the foil stemmed from the same basic category. Performance declined with an increasing number of exemplars of the same category but only by about 2% for each doubling of exemplars. Hence, also for scene memories a detailed representation of content was found. When images of the same theme were manipulated in such a degree that their similarity to the target images decreased, performance declined accordingly, indicating a visual component to image representations [17].

To summarize, results of previous studies differ in their conclusions about the memory representations created upon viewing images, and did not necessarily disentangle the contributions of visual or conceptual information. Our main aim was to address the issue how visual and conceptual information contribute to memory for images, in conjunction and separately. Participants viewed a sequence of images and had to recognize them subsequently. Importantly, the images that were used for testing were varied in their similarity to the previously seen images. They were conceptually similar, visually similar, both or neither. Our study was the first to use an objective and validated measure of image similarity, based on low level properties of the human visual system [1]. This is a new way to get around the problem of visual similarity, which has most often been dealt with on an ad hoc basis. It allows comparing memory for images that are only conceptually similar or only visually similar. It has been shown that the similarity estimated with the algorithm correlates highly with observers’ judgments of visual similarity [1].

Participants first viewed the target images and were subsequently tested on the distractor images in either a two-alternative-forced choice (2-AFC) condition (Experiment 1) or a yes - no decision condition (Experiment 2). We decided to use both versions in case procedure impacted on performance. Analyses of performance for the different types of distractors revealed to what extent conceptual and visual information were used in creating representations of the target images.

In a third experiment, we examined the stability of the memory representation. Participants were tested after a delay of about one week. This was done to understand if conceptual and visual information decayed in the same manner. Alternatively, similar as is true for fleeting memories [18], it could be that the pictorial component decays faster. Predictions would then be that after a delay, performance for only conceptually similar items should be lower after the delay as compared to immediate testing.

## Methods

### Image Selection

The images used in this study were derived from the COREL database. As a first step, for each image the visual similarity to all other images was calculated. For the development of the similarity measure, a representation of colour and spatial frequency, similar to the one implemented in the primate visual system, was used. The colour index was based on the opponent colour processes as implemented in the colour opponent retinal ganglion cells of primates. The spatial index was based on multiresolution, multiorientation frequency filters, similar to the ones implemented by simple and complex cells in primary visual cortex [1]. Important for our study, the authors showed that, to a large degree, the rank orders induced by the indexes predicted the perceived similarity between images.

The target images for the subsequent experiments were chosen by the authors of this paper. They were selected in such a way that they had a clearly nameable meaning and that possibly there would be numerous different variants of the respective image content. The images of the COREL database had been organized into categories with images of one category belonging to the same theme [19]. Then, for each target image, four distractors were chosen. The conceptually and visually similar distractor (called ‘con_vis’ hereafter) was defined as the image with the highest visual similarity, from the same category as the target. When the most similar image was only a variant of the target image, for example, the same image taken from a slightly different angle, it was not selected. Instead the next most similar image was chosen. In all cases, the con_vis distractor was among the ten most visually similar images. The conceptually similar distractor (called ‘con_only’ hereafter) was the image with the least visual similarity to the target, in the same category. When this image differed in gist from the target image, as occurred in some categories, then the image ranking next in visual dissimilarity was selected. The chosen distractor was always among the ten least similar images from the same category. The third distractor, the visually similar one (‘vis_only’ hereafter), corresponded to the image from a different category with the highest visual similarity to the target image. The only restriction with respect to choosing the image was that it had to have a nameable gist (e.g., it could not be a random pattern or structure) and that this gist was not identical to that of any other target or distractor item. The random distractor (called ‘random’) was neither conceptually nor visually similar to the target image. It corresponded to the image that was in the middle of the similarity comparisons of the target image with all other images. Again, if the image either had no gist or a gist that was equivalent to any of the other images, it was not chosen but instead one of the neighbouring images was selected.

**Figure 1:**
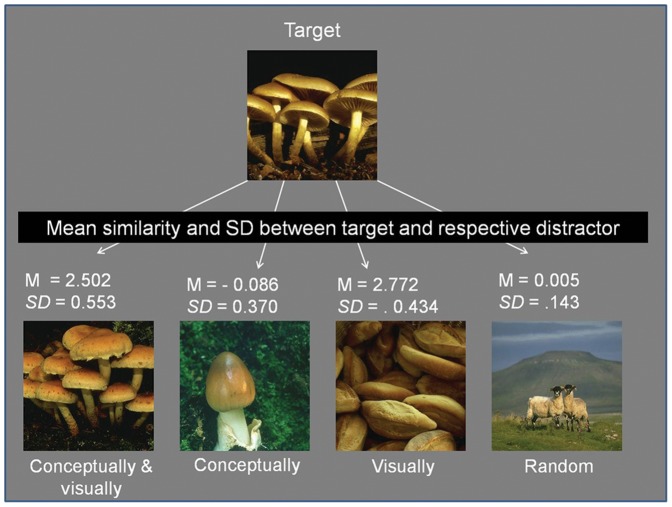
The target image is shown on the top and the four corresponding distracters on the bottom. The mean similarity and the standard deviation of the target and each distractor are shown in-between, as z-scores.

With the procedure described above, initially 59 image sets consisting of a target image and four distractors were chosen. All images were presented in randomized order to three students of the University of Giessen who were naive with respect to this study. Their task was to give a verbal label to each image in as short a description as possible. This was done to ensure that the images that were supposed to be conceptually the same would be labelled the same and that those that were supposed to be different, would be assigned a different label. 51 sets of images were kept after the validation procedure. We calculated the similarity between each target and the different distractors on the remaining data set of 51 image sets. The similarity values are given as *z*-scores where a value of 0 corresponds to the mean. As can be derived from what is stated above, the target image should be highly visually similar to the con_vis distractor and to the vis_only similar distractor. The similarity between the target and the con_only distractor and the random distractor should be in the medium range in the *z*-values of similarities. Figure 1 shows an example of an image set with the target depicted on the top and the four distracters on the bottom. The mean similarity between target and each distractor is also indicated.

The mean similarity between the target and the con_vis distractor was *M* = 2.502 (*SD* = .553). The mean similarity between target and the con_only distractor was *M* = −.0860 (*SD* = .370). The mean similarity between the target and the vis_only distractor was highest with *M* = 2.772 (*SD* = .434). For the random distractor, the mean similarity was *M* = .005 (*SD* = .143).

A repeated measures ANOVA was conducted to test for a significant effect of the distractor class on the target-distractor-similarity The main effect of distractor type was highly significant, *F*(3, 150) = 881.702, *p*<.001. All pairwise comparisons with Bonferroni correction were highly significant (all *p*≤.003), except the comparison between the con_only and the random distractor.

We selected another 168 images. They were to be included in the image sequence that participants had to memorize but no testing occurred for these images. By placing half of the foil images before and the other half after the target images in the sequence, we wanted to prevent any potential primacy or recency effects with respect to memorization of targets. The foil images were conceptually and visually dissimilar to all target images. The mean of the averaged visual similarity between each target and the foil images was *M* = −.0895 (*SEM* = .03). The similarity between the targets and the foil images was significantly lower than both the similarity between the target and the con_vis distractor and the similarity between the target and the vis_only distractor (both *p*<.001).

As reported above, both the con_vis distractor and the vis_only distractor were highly visually similar to the target whereas the other two were not. Estimated visual similarity has been shown to correspond well to subjectively perceived similarity [1]. Thus, initially, we did not additionally validate the similarity by having human observers make judgements between images from different categories. However, we opted for an additional validation after initial data collection to address the potential criticism that the images chosen based on calculated similarity would not correspond to human similarity judgements. We report this validation procedure in Appendix A.

### Participants

16 students participated in Experiment 1 and Experiment 2, respectively. In Experiment 3, 12 students participated. All of them had self-reported normal or corrected-to-normal vision and only participated in one of the experiments. They were all students of the Justus-Liebig University Giessen and received 4 Euros for their participation. All were naive with respect to the purpose of the study.

#### Experimental equipment

The experiment was written in Matlab, using the Psychophysics Toolbox [20], [21]. The experiment was run on Microsoft Windows XP. Images were presented on an Iiyama VisionMaster 513 (MA203DT) 21″ CRT screen. Monitor resolution was 1280×960 pixel. The refresh rate was 100 Hz. A chinrest to stabilize head position of the participants was placed at a distance of 47 cm from the screen, resulting in a visual field of 48.2° by 36.9°.

### Procedure

#### General information

Participants were told that they would first, for about 15 minutes, see a sequence of images that would be presented subsequently at the centre of the computer screen. They were instructed to memorize all images in preparation for a recognition task. The lighting conditions were kept constant across participants. The experimenter stayed in the lab room during the experiment. The experiment was undertaken with the understanding and informed consent of each subject. We strictly followed the guidelines of the German Psychological Association. These guidelines are quite similar to those of the American Psychological Association (APA) and can be found here: http://www.dgps.de/dgps/aufgaben/003.php (see paragraph C.III). All observers were read detailed instructions at the beginning of the experiments according to the ethics guidelines of the German Association of Psychology (ethics board of the Deutsche Gesellschaft für Psychologie, DGPs: http://www.dgps.de/dgps/aufgaben/ethikrl2004.pdf). The experiment was only started after observers consented to these conditions. The instruction sheet serves as documentation for their informed consent. The experimental protocol conformed to the Declaration of Helsinki and the ethics guidelines of the German Association of Psychology and did thus not require any additional ethics approval. All data were analyzed anonymously.

#### Study phase

Participants were seated in front of the monitor. After the verbal instruction, the experimenter started the first phase of the study. In this learning phase, participants viewed a sequence of 219 images. Presentation time of each image was 750 ms. The images were presented in colour at the centre of the screen, and were separated by a blank interval of 1000 ms. Image size was 15 degrees of visual angle. The learning phase lasted about 15 minutes. As described above, the image sequence was constructed in such a way that the first 84 images were foil items; then the 51 targets were shown, followed by the other 84 foil images. Participants did not know about this partition; the instruction was to memorize all images. The order within these three subdivisions was randomized for each subject. Which half of the foil items appeared before or after the target images, was counterbalanced across subjects. After image presentation, a five minute break was taken. During this time, the experimenter talked to the participant about task-irrelevant matters to prevent the subject from actively rehearsing the study material. In Experiment 3, subjects were told before viewing the image sequence that the recognition task would take place after one week.

#### Test phase

In the test phase of the first experiment, participants performed a 2-AFC recognition task. Upon a key press by the participant, two images, the target and one distractor, appeared next to each other on the screen. The images remained visible for 2000 ms. There was no time limit with respect to giving the answer. Each target appeared equally often on the left and the right side. Participants pressed the left and right arrow keys to choose the image on the left or the right of the screen as the target image. The order of the test trials was randomized. For every subject, there were 13 or 14 trials for each of the four distractor-target combinations, resulting in a total of 51 trials. The number of trials for the respective distractor types was counterbalanced across participants. Each target image appeared equally often with each of the four possible distractor images across participants.

The test phase of the second experiment differed from the one of the first in that only one image was shown. It was also visible for 2000 ms, and participants had to decide whether it had been present in the previous presentation phase of the images. They used the left arrow to indicate “yes” and the right arrow to indicate “no”. In about half of the trials (either 25 or 26), the target was shown. In the other half, a distractor from one of the four different categories was shown. Again, the number of trials from the respective distractor categories was counterbalanced across participants.

The test phase for the third experiment was identical to the test phase of the first experiment, that is, a 2-AFC recognition task.

## Results

An alpha level of .05 was used for all statistical tests. Posthoc pairwise comparisons were Bonferroni-corrected. The reported *p* values correspond to the exact *p* values.

### Experiment 1

#### Accuracy in the memory task

**Figure 2:**
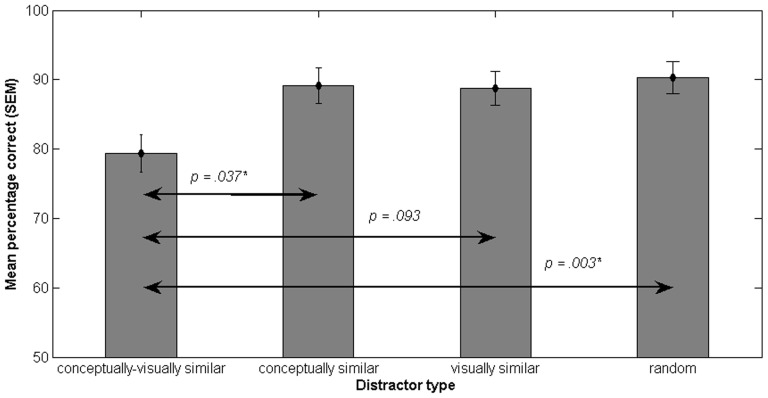
Mean percentage correct and SEM (y-axis) for the four target-distractor pairings (x-axis), Experiment 1. The *p*-values resulting from the posthoc pairwise comparisons of the ANOVA are indicated above the arrows, with a star denoting a significant difference.

Memory performance was defined as the percentage of correct answers in the memory test. We compared accuracy between the four different target-distractor-combinations. Figure 2 shows performance as the mean percentage correct and the standard error of the mean (SEM) for the four distractors types. Performance was lowest when the distractor had been conceptually and visually similar to the target, *M* = 79.39% (*SD* = 10.80). Performance in the other three conditions was very similar (for the con_only distractor: *M* = 89.14%, *SD* = 10.50; for the vis_only distractor: *M* = 88.76%, *SD* = 9.98; for the random distractor: *M* = 90.25%, *SD = *9.25).

A repeated measures ANOVA showed a significant main effect of distractor type on performance, *F*(3, 45) = 7.274, *p = *.002. Pairwise comparisons with Bonferroni adjustment showed that the difference between the con_vis and the con_only distractor was significant (*p* = .037) as was the difference between the con_vis distractor and the random distractor (*p* = .003). None of the other differences were significant.

Another way of analysing the data is by using a repeated measures ANOVA with two factors (conceptual similarity, visual similarity) with two levels (high, low). The main effect of conceptual similarity was significant, *F*(1, 15) = 8.493, *p* = .011. Performance was lower for higher conceptual similarity (estimated marginal means: 84.26 for high conceptual similarity and 89.51 for low conceptual similarity).

The main effect of visual similarity was also significant, *F*(1, 15) = 13.95, *p* = .002. Performance was lower for higher visual similarity (estimated marginal means for high visual similarity *M* = 84.08 and for low visual similarity *M* = 89.70). The interaction effect was not significant; *F*(1, 15) = 3.44, n.s.

### Experiment 2

The second experiment differed from the first experiment only insofar that a “yes-no” recognition procedure was used in the test phase.

#### Accuracy in the memory task

**Figure 3:**
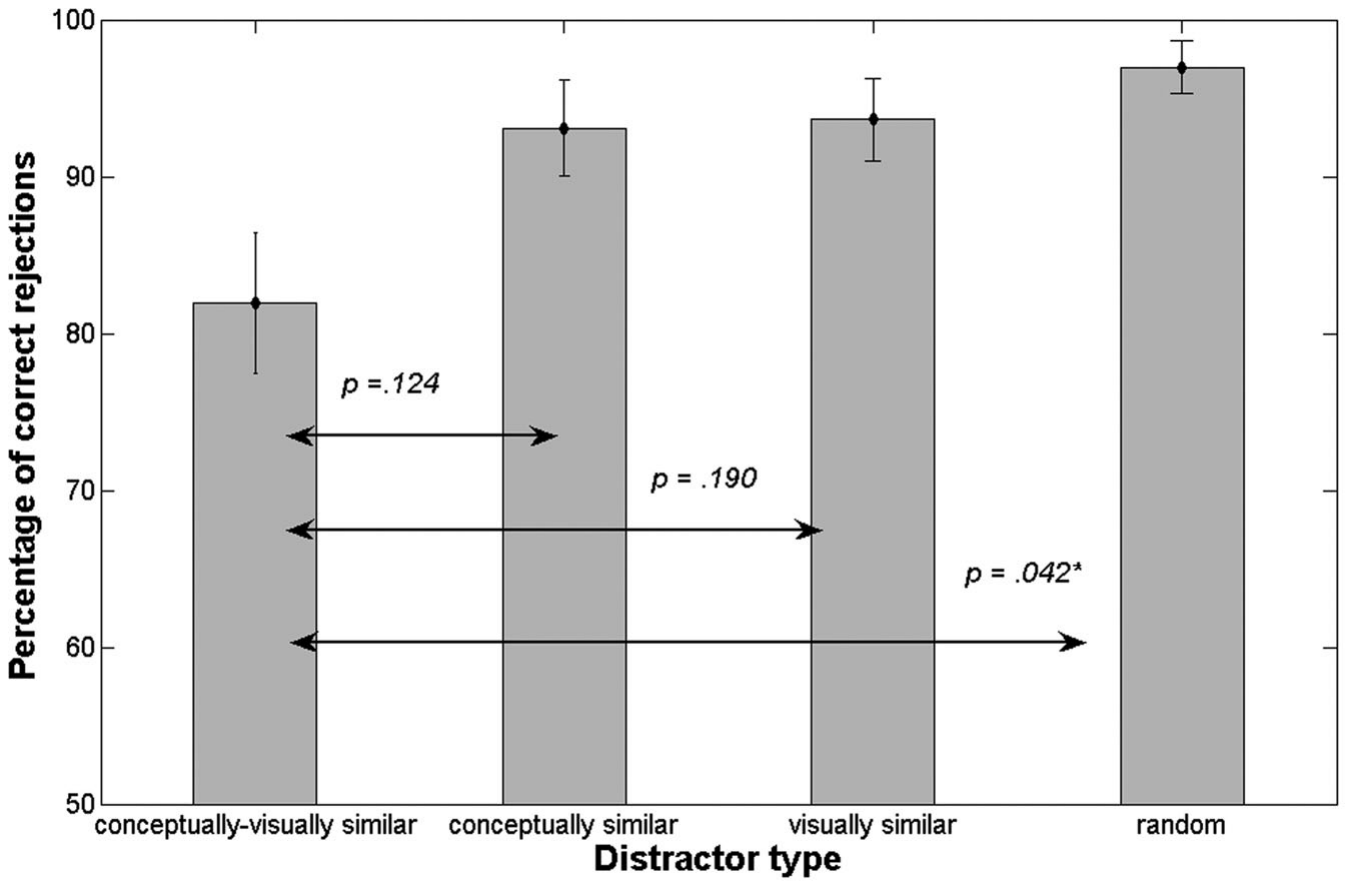
Mean percentage of correct rejections and SEM (y-axis) for the four target-distractor pairings, Experiment 2. The *p*-values resulting from the posthoc pairwise comparisons of the ANOVA are indicated above the arrows.

The mean hit rate, that is correct identification of the target, was *M* = 83.69% (*SD* = 12.45). The performance for the four distractor classes was of greater interest for our study; hence, we compared the values of correct rejections. Figure 3 shows the mean percentage of correct rejections and the SEM for the four distractors types. Performance as correct rejections was worst for conceptually and visually similar images with a mean of *M* = 81.96% (*SD = *17.99). Thus, in about 20% of the trials, participants mistook the con_vis distractor for a previously seen image. The mean percentage of correct rejection of con_only distracters was *M* = 93.10% (*SD* = 12.20) which was about the same as for vis_only distractors (*M = *93.63%, *SD = *10.55). For the trials when a random distractor was presented, the rate of correct rejection was very high with a mean of *M* = 96.96% (*SD = *6.64).

A repeated measures ANOVA showed a significant main effect of distractor type on the rate of correct rejection, *F*(3, 45) = 4.88, *p = *.010. Pairwise comparisons with Bonferroni correction showed that the difference between the rate of correct rejection for the con_vis and random distractor was significant, *p = *.042. Performance for the con_vis distractor was not significantly different from performance for the con_only distractor and the vis_only distractor.

Again, the data can be looked at by taking conceptual and visual similarity as two factors. There was a significant main effect of conceptual similarity, *F*(1, 15) = 4.85, *p* = .044. Performance was lower for higher conceptual similarity (estimated marginal means M = 87.53% for high conceptual similarity and M = 95.30% for low conceptual similarity). The main effect of visual similarity was also significant, *F*(1, 15) = 5.81, *p* = .029. Again, higher similarity was associated with lower performance (estimated marginal means *M* = 87.80% for high visual similarity and *M* = 95.30% for low visual similarity). The interaction was not significant, *F*(1, 15) = 3.21, n.s.

**Figure 4:**
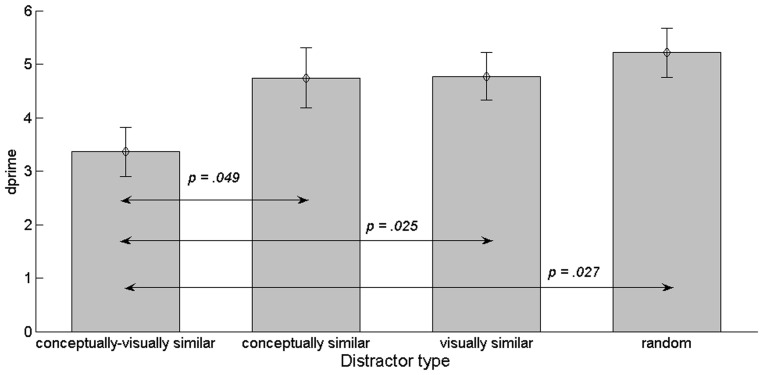
d’ prime and SEM for the four-target distractor pairings, Experiment 2. The *p*-values resulting from the posthoc pairwise comparisons of the ANOVA are indicated above the arrows.

We used the hit rate as means of measuring performance to facilitate comparison the first experiment which had also used a percentage of correct answers. However, to consider sensitivity, i.e., to take hit rate and false alarm rate info account, we repeated the analysis based on d’(compare Figure 4). A repeated measures ANOVA on d’ values for the four image classes showed a significant main effect of distractor type, *F*(3, 45) = 5.64, *p* = .005. Posthoc pairwise comparisons showed that d’ for the class of conceptually and visually similar images was lowest than all other image classes (compared to con_only: *p* = .049, M_d_ = −1.39; for vis_only: *p* = .025, M_d_ = −1.41; for random: *p* = .027, M_d_ = −1.86). None of the other comparisons were significant. Hence, analysis of d’ led to the same finding of worst performance for the visually and conceptually similar items.

### Performance in Terms of Stored Objects

We translated the performance of the memory task into an estimate of memory capacity in terms of stored items. In Experiment 1, we took the average of the performance measure for the four distractor types. In Experiment 2, we first averaged the rate of correct rejections for the four distractor types and then averaged this value and the averaged hit rate.

To estimate the number of images held in visual memory, we first corrected for guessing, by applying the following formula: *p* = (*x* - *g*)/(1 - *g*) where *x* is the raw proportion correct, *g* the guessing probability (here corresponding to 0.5), and *p* the corrected proportion correct [22]. The result *p* was then multiplied with the number of images presented, that is, 219. For Experiment 1 this calculation led to an estimate of 163 stored images, and of 165 stored images for Experiment 2.

### Experiment 3

In the third experiment, testing occurred one week after the images had been viewed. The test phase consisted of a 2-AFC recognition task.

#### Accuracy in the memory task

**Figure 5:**
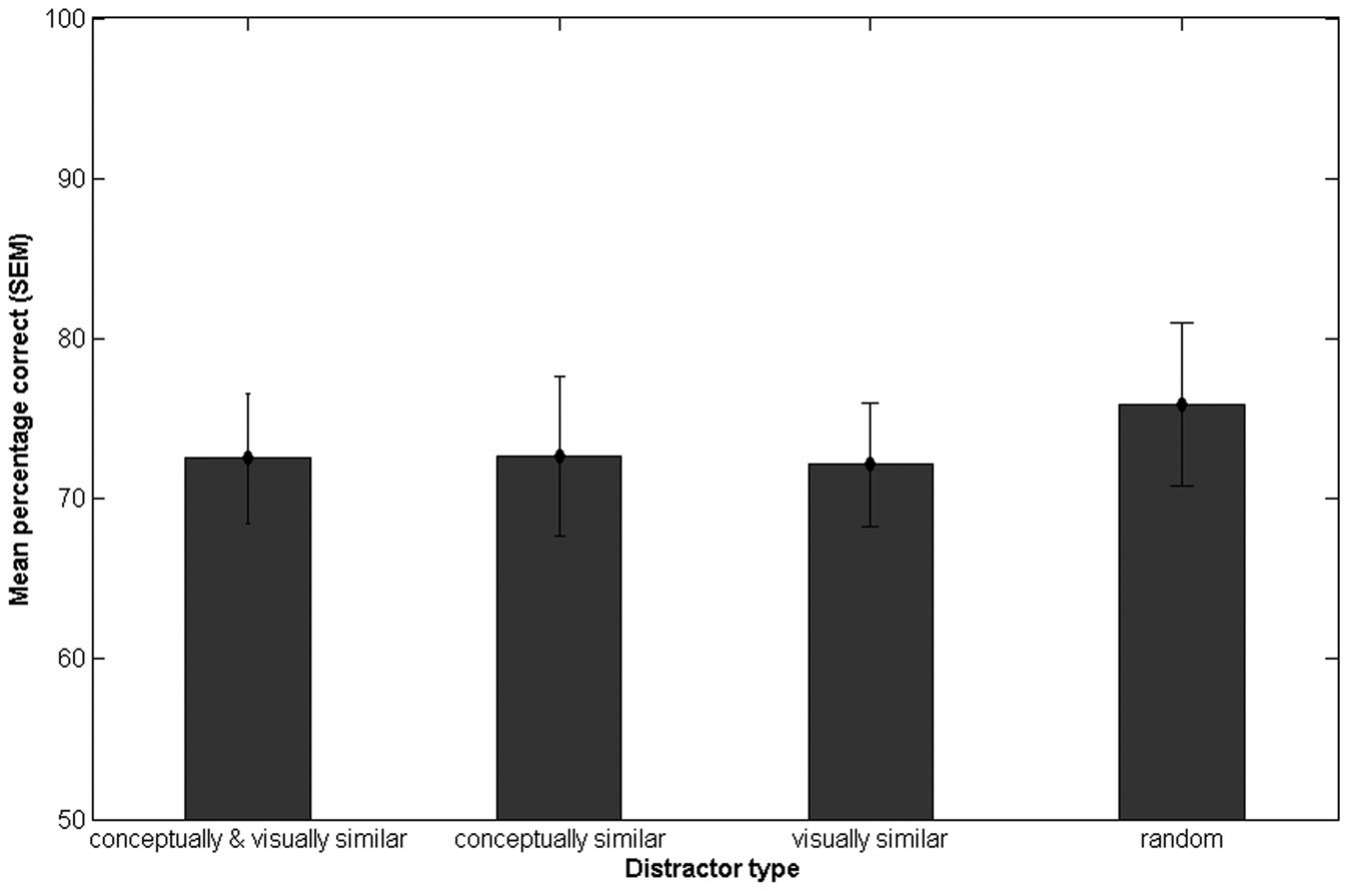
Mean percentage correct and SEM for the four target-distractor pairings when testing occurred one week after initial viewing, Experiment 3.

Performance was very similar in all four conditions. When the distractor was conceptually and visually similar to the target image, the percentage correct was M = 72.52% (SD = 14.02). For the con_only distractor, it was M = 72.65% (SD = 17.17), and for the vis_only M = 72.12% (SD = 13.31). For the random distractor the mean percentage correct was M = 75.89% (SD = 17.68). Figure 5 shows the mean performance and the SEM for the four distractor types.

A repeated measures ANOVA showed that the main effect of distractor type was not significant. When analyzing the data using a repeated measures ANOVA with the factors conceptual similarity and visual similarity, neither the main effects nor the interaction effect was significant.

The results showed that when testing occurred with a delay of one week after the images had been seen, all differences in performance between the distractor types disappeared. The mean performance corresponded to the storage of 105 images.

#### Comparison between immediate and delayed testing

We compared performance from Experiment 3 with the data from the first experiment. We calculated Welsh-tests to compare performance after immediate and delayed testing for the four distractor types. For the con_vis distractor, performance did not differ significantly. In the other three comparisons, the difference between delayed and immediate testing was significant; for the con_only distractor: *t*(26) = 2.939, *p* = .009; for the vis_only distractor: *t*(26) = 3.634, *p* = .002; for the random distractor: *t*(26) = −2.564, *p* = .021. Thus performance decreased in three conditions with delayed testing: for the con_only, the vis_only, and the random distractor, but stayed the same for the con_vis distractor.

## Discussion

In our study, we examined the role of the conceptual and visual similarity between a target and distractor image on performance in a memory task. To do this, we created sets of images where for each target four distractors were selected. One was conceptually and visually similar, one only conceptually similar, one only visually similar and the fourth one neither conceptually nor visually similar to the target image. Importantly, visual similarity was estimated by applying an algorithm combining information on the distribution of colour and spatial frequencies. This approach overcomes the problem of how to define visual similarity. After having viewed the images, participants performed a 2-AFC recognition task (Experiment 1) or a yes - no decision on a single image (Experiment 2). In Experiment 3, testing occurred with a delay of one week, using the 2-AFC procedure. In the first two experiments where testing occurred subsequent to image viewing, performance was worst for distractors that were conceptually and visually similar to the target. Repeated measures ANOVA showed significant main effects for both conceptual (high vs. low) and visual similarity (high vs. low) showing that both factors matter.

Performance differences depending on distractor type disappeared when testing occurred after a delay of one week: performance decreased in the delay-condition for all distractor types except for the con_vis distractor. This indicates that memory decay occurs both on the conceptual and visual dimension. If only decay of visual information occurred, then participants should have made more errors in the delayed condition in trials with a conceptually similar distractor than in trials showing a random or a visually similar distractor. If only conceptual information decayed, then performance should have been lower for the visually similar distractor. However, the drop in performance was alike across distractor types.

How can it be explained that there is no decrease in performance for the con_vis distractor as compared to the immediate delay? It could be that with immediate testing, there is a high level of interference during testing when two highly similar images are seen before the target image has been consolidated properly. With a delay of one week, there would be no such interference anymore because the memory would have been consolidated in depth over the delay period. Alternatively, it could be that for the other distractor types interference causes the decrease in performance because in the course of a week, much additional conceptually and visually similar input is encountered. For the conceptually and visually similar distractors, maximum interference would have already occurred after testing; no further decline would take place.

Across conditions, the general level of performance was remarkably high. From this, two main conclusions can be drawn: (1) Both conceptual and visual features are extracted when viewing images and memorizing them. This is reflected in the lower performance for those trials when the distractor image was similar on both dimensions to the target image and by the main effects of both factors. (2) Visual long-term memory is amazingly good and detailed with respect to visual information.

According to the Dual Coding Theory [23]–[25], upon viewing an image, an observer creates two traces, a visual and a verbal one. These two traces are additive and hence improve memory performance. Applied to the present study this means that memory performance should be worse when the target image is similar to the distractor image on both dimensions, e.g. conceptually and visually similar, exactly what was found. The fact that no performance differences between the other three distractor types were found is likewise reconcilable with the Dual Coding Theory: The traces are believed to be independent; and hence similarity between target and distractor image on one dimension, i.e. visual or conceptual similarity, would still allow correct differentiation based on the second trace.

Still, a replication of the present study with more difficult conditions is desirable in order to test if the non-existing differences for the random, conceptual, and visual distractors are due to ceiling effects; i.e., the experiment being too easy to detect any differences.

We found no differences in performance between only visually and only conceptually similar images which could indicate a similar contribution of both type of information in creating memory presentation. It has previously been reported that conceptual distinctiveness was of greater importance than perceptual distinctiveness [26]. One likely explanation why we had not found worse performance for conceptually similar than visually similar performance, as expected from the finding cited, is simply that in our study only one image was similar the target image but not that multiple versions of one scene had to be held in memory. Conceptual distinctiveness may only play a role when multiple images of one gist have to be remembered. Recognition memory is thought to be supported by the underlying processes of recollection, i.e., remembering an occurrence, and familiarity, a ‘know’ feeling (for a review, see [27]. A 2-AFC task is supposed to rely more on familiarity; i.e., the participant can compare the relative familiarity of both alternatives and choose the item that is more familiar. A yes-no task supposedly relies on recollection meaning the information associated with an item needs to be recalled [28]. Neuroanatomical differences for those subsystems have been found [29]. We have found the same pattern of results - worst performance for conceptually and visually similar distracters - with both procedures. Assuming that a division into those two processes is valid (for contradicting findings, compare [28], [30]), then our data indicates that both recollection and familiarity are affected by the similarity between target image and distracters image.

While some theories claim that there is no need for visual memory as we can just use the world as an external memory [31], [32], our data is in line with a large body of evidence supporting the notion that lasting, detailed representations are created (e.g., [14]–[17], [33], [34]). If one assumes that storing visual details is higher in cost than only storing the gist, what is the additional advantage of remembering details? First of all, one needs to consider that it might not have high costs. It has been shown that visual long-term memories are created incidentally [35]. If detailed representations result of a side-product of normal looking at scenes, then there are no additional costs for memorizing the viewed material, neither with respect to its gist nor visual details.

Humans are particularly apt in remembering visual material: memory is better for studied pictures than for studied words, the picture-superiority effect [23], [24], [36]–[38]. The general explanation for this effect is that for pictorial input two traces are created, a visual one and a verbal one whereas verbal stimuli do not automatically generate a visual trace but only a verbal one [25]. In visual search tasks, detailed visual information proves of advantage: Search times within repeated scenes decreased across repetitions and recall for target location was superior for repeated scenes [39]. Such a mechanism would be of great advantage when re-encountering situations that call for quick actions. Models of viewpoint-dependent object recognition rely on the assumption that information specifically corresponding to discrete views form the basic representational structure of objects (e.g., [40]–[42]); a long-term memory high in capacity and visual detail indicates that such a mechanism of object recognition is feasible. To summarize, the creation of visual memories from visual input might come at no cost or at a lower cost than creating a comparably detailed representation in a different modality.

### Conclusions

We showed that participants extracted and remembered both conceptual and visual information when looking at natural scenes in preparation for a memory task. When testing occurred after a delay of a week, performance was the same for all distractor classes, signifying decay both of conceptual and visual information. In general, performance was remarkably high, indicating a long-term memory that is large in capacity and high in detail. This might have proven of advantage in the evolution of our species. Furthermore, this has implications for models of our visual system, for example, one might infer that view-dependent models of object recognition are feasible.
